# A Scalable System for Passively Monitoring Oral Health Behaviors Using Electronic Toothbrushes in the Home Setting: Development and Feasibility Study

**DOI:** 10.2196/17347

**Published:** 2020-06-24

**Authors:** Vivek Shetty, Douglas Morrison, Thomas Belin, Timothy Hnat, Santosh Kumar

**Affiliations:** 1 Section of Oral & Maxillofacial Surgery Department of Biomedical Engineering University of California, Los Angeles Los Angeles, CA United States; 2 Department of Biostatistics Fielding School of Public Health University of California Los Angles, CA United States; 3 Department of Computer Science The University of Memphis Memphis, TN United States

**Keywords:** health behaviors, oral self-care, digital tools, remote monitoring, passive measurement

## Abstract

**Background:**

Dental disease (including dental caries and periodontal disease) is largely preventable and closely linked to inadequate oral health behaviors. Digital health technologies have great potential for unobtrusively monitoring brushing behaviors in home settings and promoting optimal oral self-care routines at scale.

**Objective:**

The aim of this study is to leverage the ubiquity of electronic toothbrushes and smartphones with the development of a Remote Oral Behaviors Assessment System (ROBAS) and evaluate its feasibility for passively tracking brushing behaviors in real-world settings.

**Methods:**

We developed ROBAS by linking inertial sensors contained within consumer electronic toothbrushes to a scalable software platform comprised of a smartphone app linked to a cloud platform. First, the criterion validity of ROBAS for accurately capturing brushing details was established in a laboratory setting. Next, real-world performance and usability were evaluated in a stratified community sample of 32 participants who used ROBAS daily for 1 month and maintained a diary of their brushing episodes. Semistructured interviews at baseline and exit captured the user experience. We used regression models and Bland-Altman analyses to assess the criterion validity, functionality, accuracy, and consistency of ROBAS.

**Results:**

Using a stopwatch as the criterion reference, ROBAS showed a mean absolute percent error (MAPE) of 1.8%, an estimated bias of 0.64 seconds that was not statistically distinguishable from zero (95% CI –0.93 to 2.22 seconds, SE 0.79), and a connection failure rate of 6.7% (95% CI 0.8%-22.1%, SE 4.6%). In real-world testing, ROBAS showed close agreement with the daily diary recordings of brushing episodes; estimated average discrepancies between the diary and ROBAS were 0.13 sessions per day (95% CI 0.01-0.26, SE 0.06), 8.0 seconds per brushing session (95% CI 1.4-14.7, SE 3.3), and 30 seconds of brushing per day (95% CI 9.3-50.1, SE 10.0). Retrospective self-reports produced substantially higher estimates of brushing frequency and duration compared to ROBAS measurements. Participants reported ROBAS was easy to use and expressed an interest in receiving ROBAS-delivered feedback on their brushing behaviors. Most participants were bothered by the use of an additional study phone, and some reported connectivity-related issues.

**Conclusions:**

ROBAS has a high criterion validity for measuring oral health behaviors. It can accurately and reliably monitor brushing patterns in home settings for extended periods. Unobtrusive data collection through ROBAS sets the stage for automated coaching and optimization of oral self-care practices at the individual and population level.

## Introduction

Although largely preventable, dental disease (including caries and periodontal disease) is extremely common and exacts a substantial personal and societal toll [1,2]. Dental disease is closely linked to poor oral hygiene behaviors (OHB). Considerable scientific evidence indicates that systematic, twice-a-day tooth brushing with a fluoridated toothpaste prevents accumulation of dental plaque (a sticky film containing bacteria) that leads to tooth decay, gum disease, and eventually, tooth loss [3-5]. However, this basic health behavior is not as widely and fully practiced as dentists and health organizations would like it to be [6]. Large population surveys reveal that a majority of individuals (45% to 67%) brush less frequently than twice a day, with brushing habits worsening with advancing age [7,8].

The essential predicament of traditional dental care is that what happens during the roughly 363 days of the year that typical patients spend outside of the dental clinic is of far greater consequence than the 2 days when they have clinic visits [9]. Self-reports rarely provide the care provider with an accurate picture of actual brushing behaviors because of distorted recall of what, for the vast majority of patients, is a low-salience activity as well as associated social desirability biases [10,11]. Furthermore, oral hygiene instructions delivered during dental care visits are not scalable and do not reliably inspire lasting habits because they are very generic; sporadic; and largely disconnected from the patient’s values, needs, and preferences. Ultimately, good oral health depends on the individual’s ability and willingness to best carry out the mundane self-care behaviors at home. To achieve meaningful improvements across large, diverse populations, it is essential for providers to identify ways to extend care beyond the confines of the dental clinic and to support and reinforce optimal oral health behaviors in the home setting.

The convergence of technological advances, deep penetration of digital devices, and a generational shift in how the technology is used and consumed provides unique opportunities to connect remotely and to engage with patients at a personal level [12,13]. Self-care technologies, including devices whose embedded sensors and analytic algorithms can track, analyze, and guide the user’s behaviors, are increasingly used to help individuals recognize and improve daily lifestyle choices that add up to affect their health [14-16]. Wearable devices like the Fitbit (Fitbit Inc) and the Apple Watch (Apple Inc) are prominent examples of these self-tracking technologies. Initially developed to help users measure daily activity, they have evolved into connected health platforms that seek to facilitate healthy behaviors by providing individuals with relevant feedback, timely and personalized cues, and motivational rewards, all of which may support health behavior change.

Building on technology’s potential to cultivate interest and awareness in mundane self-care behaviors, we set about creating a low-cost digital platform that could measure and track oral health behaviors in the lived environment and set the stage for automated, personalized coaching at population scale. Such a Remote Oral Behaviors Assessment System (ROBAS) would leverage the ubiquity of electronic toothbrushes and smartphones as well as sociotechnological trends in how digital devices are used. Our objective was to develop a ROBAS and to evaluate its performance and feasibility for passive tracking of brushing behaviors in real-world settings.

## Methods

### Development of ROBAS

**Figure 1:**
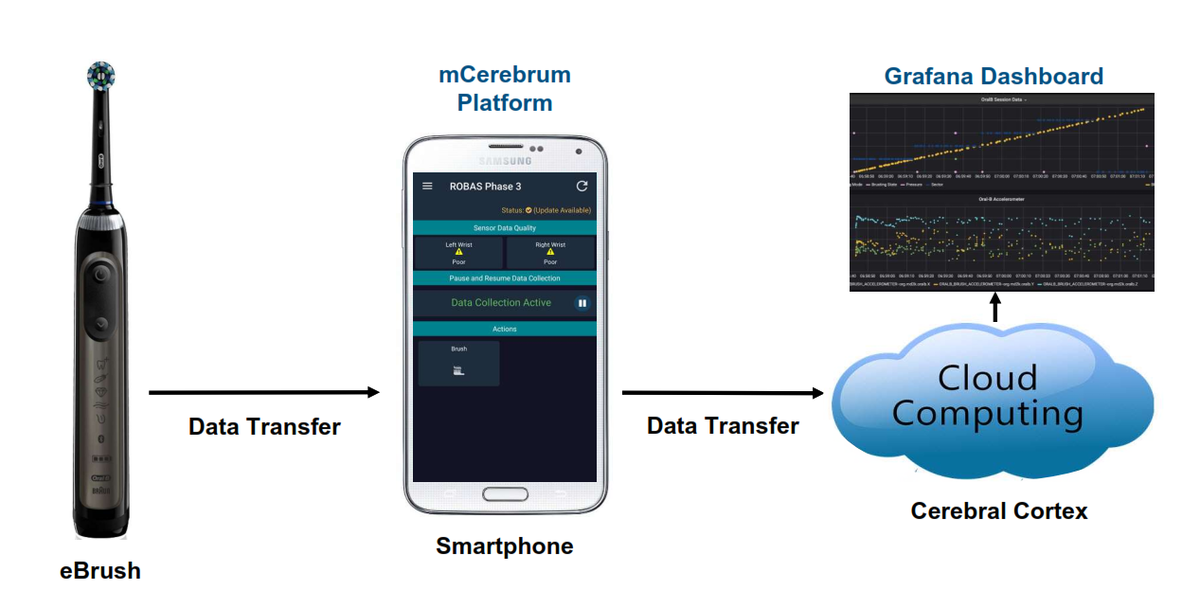
Brushing data generated by the electronic brush (eBrush) collected by a smartphone app and transmitted to a computing cloud for analysis and monitoring.

ROBAS builds on a broadly available consumer-grade electronic toothbrush (Oral-B 7000; Procter & Gamble) as the data source for brushing behaviors (timing, duration, pressure applied). With the permission of the manufacturer, the application programming interface of the Oral-B toothbrush was adapted to expose the brushing data captured by the embedded accelerometer and pressure sensor. Collected data is transmitted over BLE (Bluetooth Low Energy) to a paired Android mobile device running the companion mCerebrum data collection app [17]. Developed by the Mobile Data to Knowledge (MD2K) Center, mCerebrum is a highly extensible, open-source platform that permits concomitant data collection from multiple sensors with real-time assessment of data quality [18]. Collected data is then uploaded to the secure Cerebral Cortex cloud [19] for remote monitoring of data yields and analytics. Visualization of time series data streams of brushing episodes and monitoring of sensor function and participant compliance is accomplished through an adaptation of the open platform Grafana dashboard (Figure 1) [20].

### Research Design

The study protocol was reviewed and approved by the institutional review board, and all participants provided prior written informed consent. The first step was to verify the reliability and concurrent validity of ROBAS by comparing brushing data (duration) captured by ROBAS to data from a reference gold standard generated using a stopwatch. To assess the possibility of unacceptably poor calibration, 3 volunteer participants used ROBAS for 10 brushing sessions conducted over several days in a controlled laboratory setting. Each participant performed brushing sessions with ROBAS and a conventional stopwatch was used to record the start and stop time of each session.

Subsequently, the real-world performance and usability of ROBAS was evaluated in a community sample of 36 participants, stratified by gender, age group (18-29, 30-44, and ≥45 years), and self-reported tech-savviness (less tech-savvy or more tech-savvy). The original design contemplated balanced assignment of 3 participants to each of the 2×2×3 strata defined by gender, tech savviness and age group, with participants who dropped out early or who failed to conform to the study protocol replaced by participants from the same baseline-characteristic stratum.

In total, 2 participants dropped out at a late phase of the study and an additional 2 participants completed their exit interviews but failed to return their diaries; this left a final sample of 32 participants with 1 month of ROBAS data and diary recordings (Table 1). The balanced stratified sampling design allowed estimation of main effects as well as two-way interaction effects for the stratum-defining characteristics with a relatively modest sample size.

**Table 1:** Participants by recruitment stratum.

Age (years) and gender		Less tech-savvy	More tech-savvy
**18-29**			
	Female	3	3
	Male	3	2^a^
**30-44**			
	Female	3	3
	Male	2^b^	3
≥**45**			
	Female	3	3
	Male	2^b^	2^a^

^a^A participant completed the exit interview but did not return their brushing diary.

^b^A participant dropped out at a late stage.

At the baseline visit, participants were queried about their brushing habits including the frequency, duration, and time of day. Next, they were provided a daily diary and an electronic toothbrush paired with a dedicated Android phone (Samsung Galaxy 6). Participants were coached in the use of ROBAS by the study staff and received clarifications on its features. Participants were instructed to use ROBAS exclusively for 4 weeks at home and to time their brushing details (start time, end time, and duration) using a stopwatch and record these details in the diary.

At the exit visit, participants were asked to retrospectively estimate their brushing frequency. A brief, semistructured qualitative interview was used to assess participants’ general reactions to ROBAS, generate feedback on their experiences with the mCerebrum app, and learn about future interests in phone-based reporting of oral health behaviors. In addition to detailed observation notes by the interviewer, the interviews were recorded, anonymized and transcribed for subsequent thematic analysis. As part of the interview, participants were asked 10 multiple-choice questions with a 5-point Likert scale (Strongly agree, Agree, Neither agree nor disagree, Disagree, Strongly disagree) as response options. For easier interpretation, these answers were subsequently recoded into a favorability scale (Strongly Favorable, Favorable, Neutral, Unfavorable, Strongly Unfavorable).

### Data Analysis

All quantitative analyses were conducted using the R software system (R Foundation for Statistical Computing) [21]. We used regression models and Bland-Altman analyses [22] to assess the criterion validity, functionality, accuracy, and consistency of ROBAS. For criterion validation, discrepancies in per-session brushing duration (as recorded with ROBAS versus a stopwatch) were modeled using linear regression, with random intercepts for session nested in participant and a fixed effect for data source. For feasibility testing, brushing data (recorded with ROBAS and daily diary) were compared in terms of 3 participant-level outcomes: number of sessions recorded per day, mean per-session minutes of brushing, and mean daily minutes of brushing. Scatterplots and Bland-Altman plots were used to evaluate agreement between self-reported and measured data. The rates of reported ROBAS-related connectivity issues were also extracted from the participant diaries.

The average frequency of brushing per day was compared using values from ROBAS, diaries and self-reports provided at baseline and exit interviews. Average per-session duration of brushing was compared between ROBAS and baseline (duration was not surveyed at exit).

Per-participant scatterplots were used to explore patterns of diary recordings. The average discrepancy in per-session brushing duration among the matched sessions was estimated using a random effects model to account for repeated measurements grouped by participant.

Usability surveys were summarized across all participants, and the relationships between baseline characteristics and usability ratings were explored using univariate and multivariate linear regression models, treating ratings of Strongly Favorable as +2, Favorable as +1, Neither Favorable nor Unfavorable as 0, Unfavorable as –1, and Strongly Unfavorable as –2.

## Results

### Laboratory-Based Criterion

Each of the 3 participants completed 10 brushing sessions. In two instances, the brush failed to connect with the phone when activated, resulting in a connection failure rate of 6.7% (95% CI 0.8%-22.1%, SE 4.6%). There were thus 28 brushing sessions with data usable for analysis. Using the stopwatch recordings as criterion reference, ROBAS showed a mean absolute percent error (MAPE) of 1.8% and an estimated bias of 0.64 seconds that was not statistically distinguishable from zero; the data were not compatible with a hypothesis of a large magnitude of bias (95% CI –0.93 to 2.22 seconds, SE 0.79 seconds). Brushing durations captured by the stopwatch averaged 117.9 seconds compared to an average duration of 118.5 seconds as recorded by ROBAS. The estimated root mean squared error was 4.2 seconds.

### Performance & Feasibility Testing

#### Participant Characteristics

A total of 32 participants completed the study and provided ROBAS data as well as diary recordings of their brushing episodes (Table 1). None of the participants had previous experience with an electronic toothbrush. All reported smartphone ownership; about half the participants used their phones extensively for internet browsing, banking, and social media activities (more tech-savvy).

#### Feasibility Testing

In the home setting, ROBAS recorded 1242 brushing sessions (38.81 brushing sessions/participant) in contrast to the 1362 sessions (42.56 brushing sessions/participant) recorded in the participant diaries; a mean discrepancy of 3.75 sessions per participant (95% CI 0.19-7.31, SE 1.75), or ﻿0.13 sessions/participant/day (95% CI 0.01-0.26, SE 0.06). In total, 1095 sessions were recorded in both ROBAS and the diary, 147 sessions were recorded in ROBAS only, and 267 sessions were recorded in the diaries only (Table 2).

**Table 2:** Brushing session counts according to ROBAS and diaries.

Diary	ROBAS		
	Recorded	Not recorded	Total
Recorded	1095	267	1362
Not recorded	147	0	147
Total	1242	267	1509

**Figure 2:**
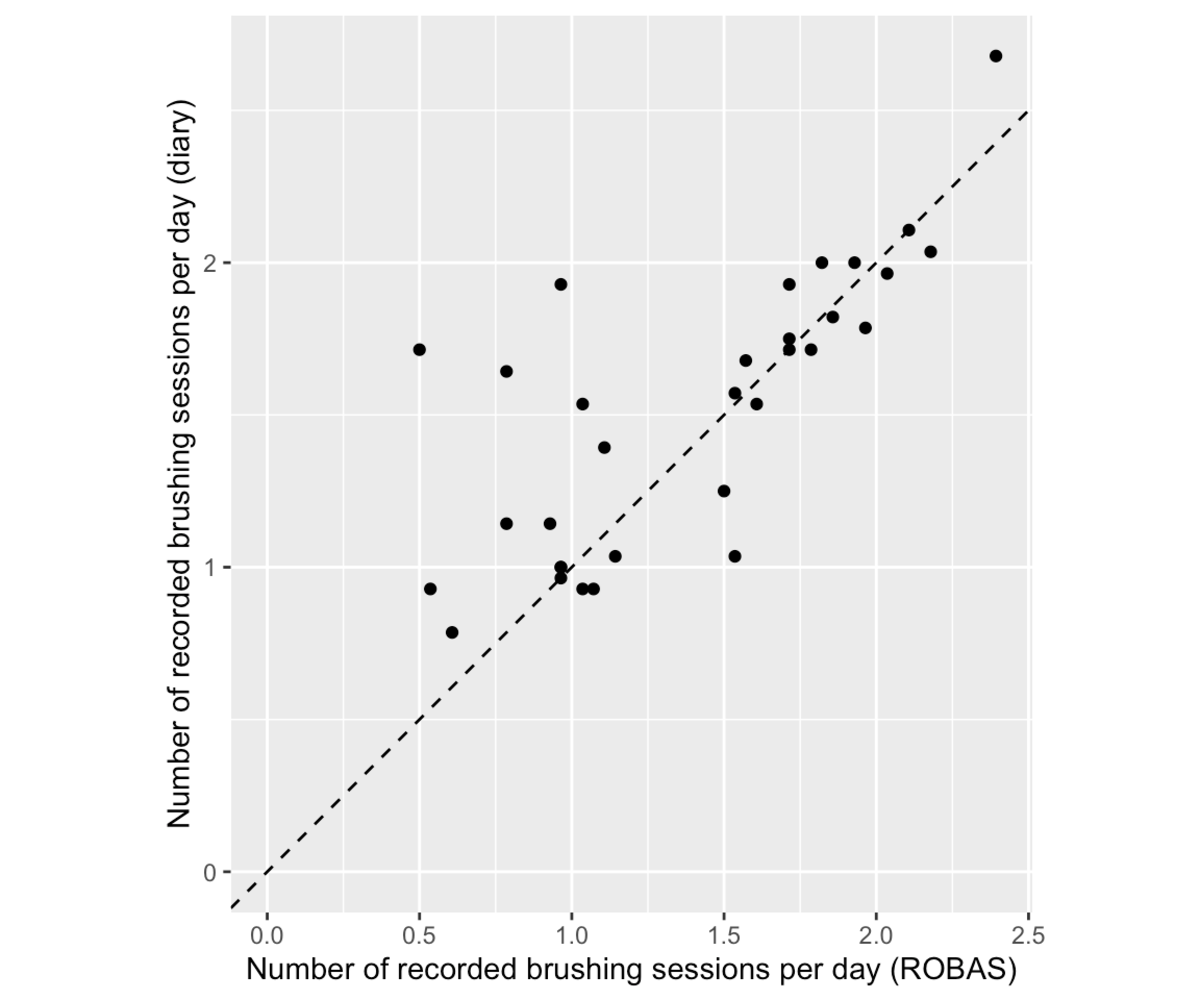
Brushing sessions recorded in participant diaries and by ROBAS. Dashed line denotes y=x. ROBAS: Remote Oral Behaviors Assessment System.

**Figure 3:**
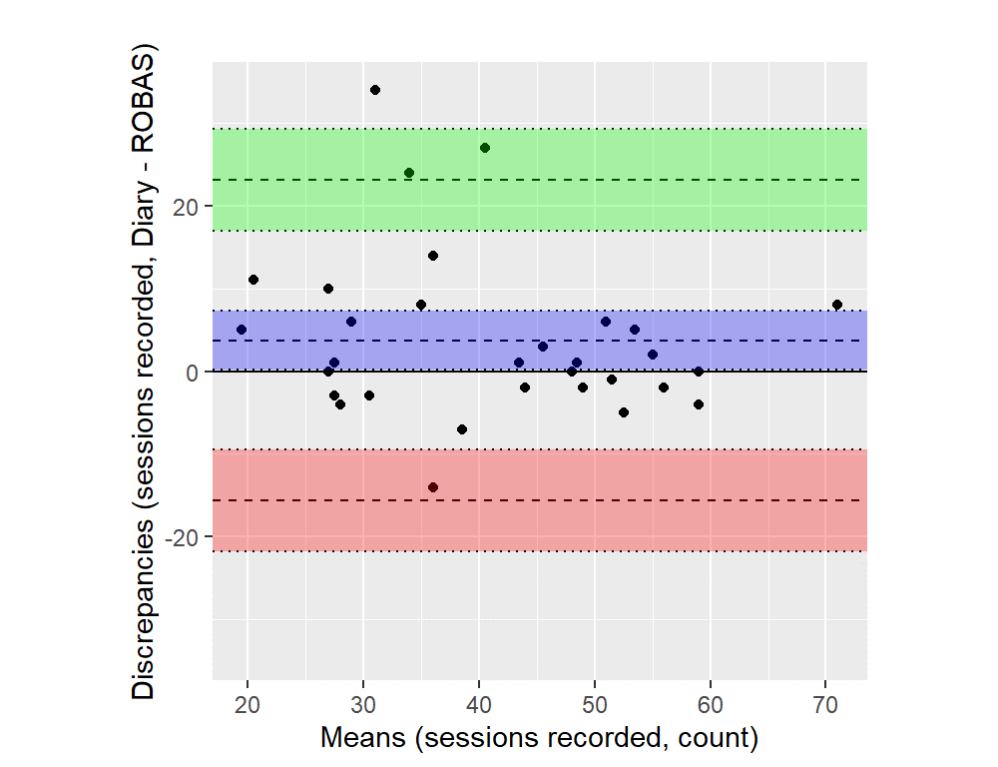
Bland-Altman plot of the number of sessions recorded, as measured by brushing diary versus ROBAS system. ROBAS: Remote Oral Behaviors Assessment System.

For the most part, the diary records of the sessions corresponded closely to that captured by ROBAS (Figure 2). However, a few participants recorded substantially more brushing sessions in the diary than in ROBAS. The Bland-Altman method was used to examine the limits of agreement between the data on brushing sessions from ROBAS and the actual sessions recorded in the diaries. The plot of this data showed an apparent relationship between the average of the two measurements and the distribution of the discrepancies; it appeared that the variance of the discrepancies was larger for participants whose measurements averaged 30 to 40 sessions, compared with participants with higher or lower averages (Figure 3). The source of this heteroscedasticity is not immediately obvious. Some of the discrepancies appeared related to technical issues; participants logged 85 instances of connectivity issues between the brush and the phone app (average 6.83%). However, the relationship between technical issues and discrepancies was not entirely clear; some of the participants with the largest discrepancies reported many technical issues, but others did not report technical issues at all (Multimedia Appendix 1).

**Figure 4:**
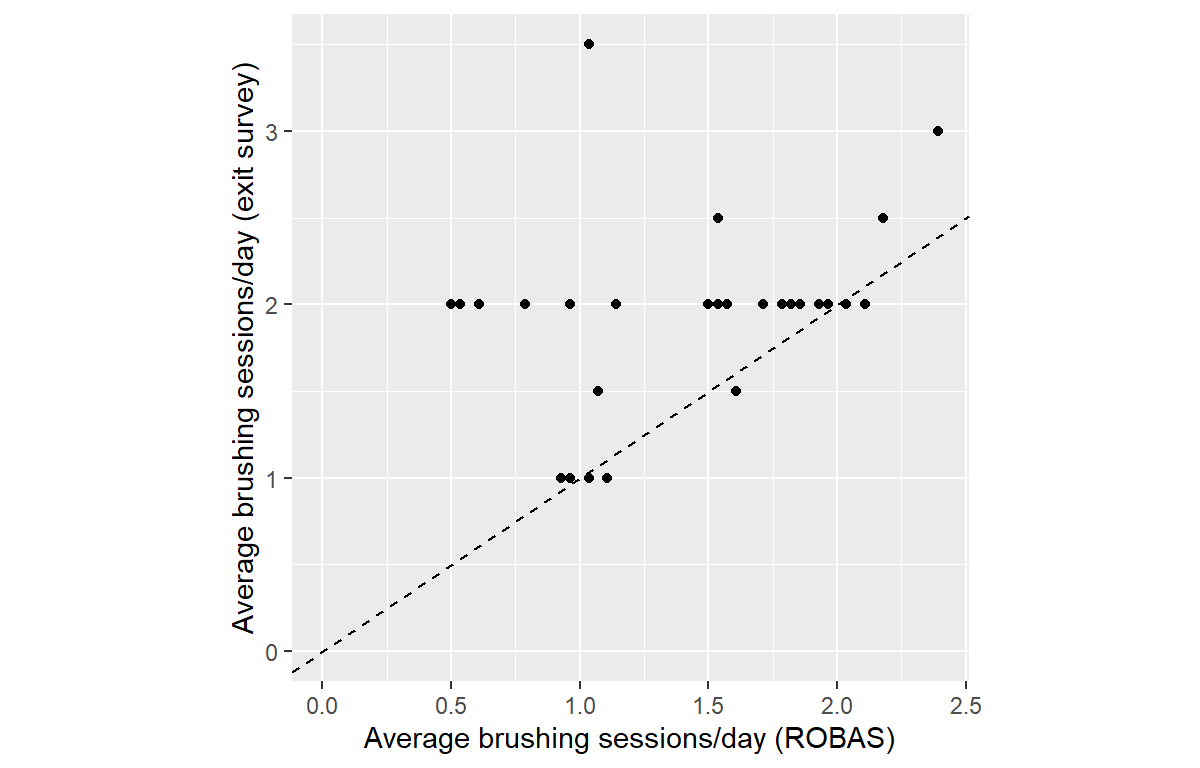
Mean daily brushing sessions by participants, as measured by ROBAS versus retrospective self-report. ROBAS: Remote Oral Behaviors Assessment System.

**Figure 5:**
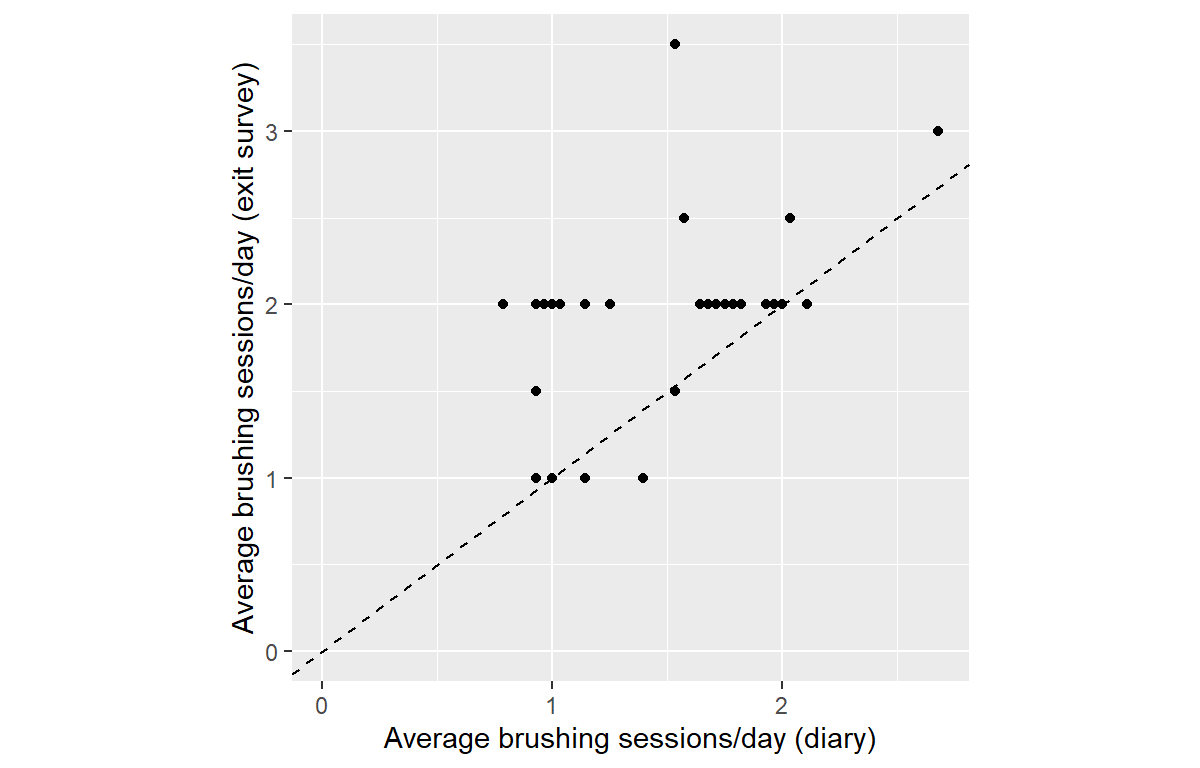
Mean daily brushing sessions, as measured by brushing diaries versus retrospective self-report.

At both baseline and exit interviews, most participants self-reported at least two brushing sessions per day. However, data captured by ROBAS data revealed that few participants actually averaged two sessions per day. Retrospective self-reports of brushing sessions/day at the exit interview were substantially higher than the ROBAS and diary data (Figures 4 and 5). On average, participants recollected at least 2.0 sessions/day (95% CI 1.8-2.1, SE 0.091). However, ROBAS documented a mean of 1.4 sessions/day (95% CI 1.2-1.6, SE 0.092) and the diaries showed a mean of 1.5 sessions/day (95% CI 1.4-1.7, SE 0.082). The estimated mean discrepancy between retrospective self-report and ROBAS was 0.57 sessions/day (95% CI 0.35-0.79, SE 0.11), and the estimated mean discrepancy between retrospective self-report and the diary was 0.43 sessions/day (95% CI 0.25-0.62, SE 0.09).

### Brushing Duration Per Session

The mean discrepancy between ROBAS and diary recordings of brushing duration/session was 8.0 seconds (95% CI 1.4-14.7, SE 3.3); ROBAS recorded an average per-session brushing duration of 137.41 seconds (95% CI 123.37-151.46, SE 6.89), whereas the diaries showed a duration 145.44 seconds (95% CI 131.12-159.76, SE 7.02). This discrepancy may be artificially small because of the unintended display of the recorded brushing duration on the ROBAS user interface.

### Individual Brushing Sessions

**Figure 6:**
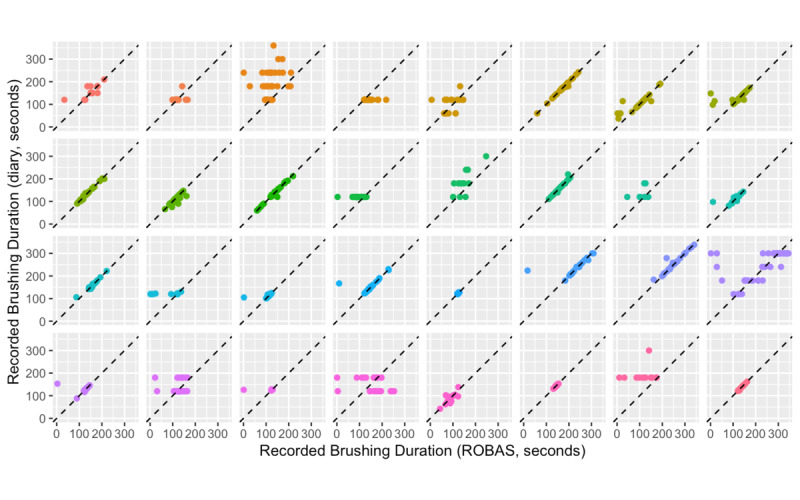
Durations of individual brushing sessions, as measured by ROBAS and brushing diaries, grouped by participant. Horizontal alignment of some data points suggests guessed approximations. ROBAS: Remote Oral Behaviors Assessment System.

The mean daily brushing duration recorded by ROBAS was 3.14 minutes (95% CI 2.65-3.63, SE 0.239), whereas the mean daily brushing duration recorded in the diaries was 3.64 minutes (95% CI 3.15-4.12, SE 0.237). The mean discrepancy between the diary and ROBAS was thus 30 seconds (95% CI 9.3-50.1, SE 10.0). Most of the individual brushing sessions recorded by ROBAS and the diaries were in close agreement (Figure 6). Among the matched sessions, the estimated average discrepancy in session duration between the diary and ROBAS was 8.6 seconds (95% CI 2.3-15.0, SE 3.2); 39% of the matched records had exactly the same elapsed time recorded in both ROBAS and the diary. Again, this close concordance may be due to the user interface design, in which the ROBAS app showed the time elapsed.

**Figure 7:**
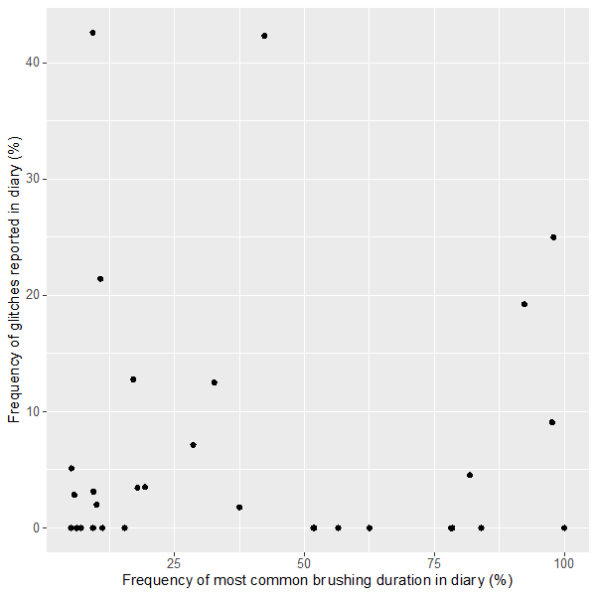
Frequency of glitches reported in diary versus frequency of most common brushing duration (eg, 2 minutes).

Several diaries showed evidence of approximated or rounded records suggesting that the entries were estimated much later than the actual event; 4 participants recorded the same brushing duration (eg, 2 minutes) for most (>90%) of their brushing sessions, indicating that the diary entries were not made contemporaneously. There was no apparent relationship between the tendency to report the same brushing duration and the frequency of reporting glitches (Figure 7).

### Usability Surveys

**Figure 8:**
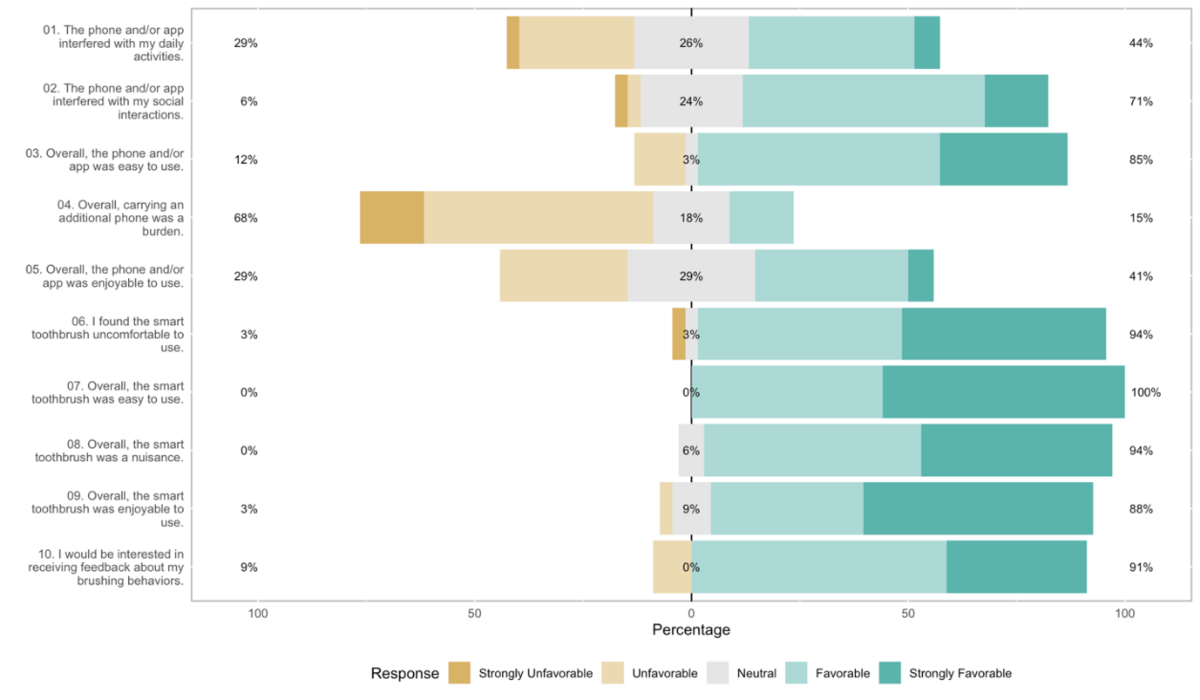
Exit interview usability survey responses; the percentages of negative, neutral, and positive responses are printed on the left, center, and right, respectively, of each color bar.

From a quantitative standpoint, satisfaction with ROBAS was uniformly high (Figure 8). Most participants (85%) found the system relatively easy to use and enjoyed the experience of the electronic toothbrush. A majority expressed an interest in receiving feedback about their brushing behaviors (91%) and indicated that they would recommend ROBAS to a family member or friend (86%). When the exit interview responses were stratified by each of the baseline characteristics, none of the questions showed evidence of differences in mean by gender, age group, or tech-savviness (a=.05). However, a sizeable segment (68%) reported that they found the additional dedicated study phone to be burdensome. Some participants (6.8%) reported technical issues involving connectivity (eg, participant was unable to connect the brush to the phone app, lag in connection) or were bothered by a short battery life.

## Discussion

### Overview

Our goal was to develop and evaluate a system (ROBAS) that would help expand oral care beyond the confines of the dental clinic. Our study showed that ROBAS is accurate and reliable in its ability to passively capture oral self-care practices (when, how long, sessions per day) in the home setting. Specifically, ROBAS (1) had very high criterion validity and demonstrated close agreement with stopwatch measurements of brushing sessions; (2) was less burdensome and more reliable than daily diaries; (3) provided a more objective and accurate representation of brushing behaviors than retrospective end-of-study recall; and (4) was generally well accepted by its users.

In a controlled laboratory setting, the details of the brushing sessions acquired by ROBAS closely approximated the ground truth values captured by the stopwatch. The high overall aggregated accuracy (<2% MAPE) and the good repeatability across brushing sessions showed that the automated data collected by ROBAS was equivalent to the manual stopwatch recordings. The concordance between the brushing session details captured by ROBAS and the daily diary records largely held up in the home setting. However, the per-session and per-day brushing durations estimated by the diaries and ROBAS showed modest discrepancies. The inconsistencies likely arose from two sources: (1) differences due to reporting errors, and (2) differences arising from technical issues.

The patterns of overreporting manifest in the diary logs of a few participants suggested a degree of fabrication with several diary entries revealing approximations of brushing duration. Some of the participants recorded “2 minutes” for every brushing session, indicating that the diary entries were not made immediately after the event. These findings underscore the challenges of depending on the willingness of participants to meticulously record details of fixed events for extended periods of time. Diary data can be of questionable reliability because they are burdensome to gather, often incomplete because of illegibility or loss, and susceptible to invention [23]. Similarly, the retrospective recall of brushing sessions/day at the exit interviews were substantially higher than that captured by ROBAS and the diary. Our results highlight the unreliability of self-reports when it comes to the recall of health behaviors of low salience [24-26]. Inaccurate estimates, originating from recall bias or social desirability bias, can lead to underestimation of risk parameters during routine dental care visits and result in erroneous self-care recommendations [27].

Technical issues related to the ROBAS prototype also contributed to an underrecording of brushing events. The glitch rate (approximately 6.8%) encountered could be related to a number of factors including failure of the toothbrush to connect to the mCerebrum app, excessive battery drain, or the participant forgetting to charge the study phone. Connectivity may have suffered from the fact that the proprietary bridging software provided by the manufacturer (Oral-B/Procter & Gamble) was written by a third-party vendor and prevented the mCerebrum app from fully controlling the brush connection process and hindered the identification and correction of root causes of connectivity failures. As our experience showed, integrating software for Android phones to communicate with a specific device’s Bluetooth stack at a low level is a process fraught with frustration as developers can implement their own custom Bluetooth interface. Resetting the devices usually reestablished connectivity; however, acceptance of any self-care technology would drop sharply if the end user is required to take frequent corrective actions [28]. Iterative versions of ROBAS have improved the connectivity issue. Additionally, low battery levels now trigger alerts to remind users to proactively charge the device's batteries.

From a usability and acceptance standpoint, participants were generally very satisfied with ROBAS and enjoyed the new experience of an electronic toothbrush. Most expressed an interest in receiving brushing summaries along with personalized actionable suggestions. Interestingly, the participants did not express any concerns about privacy issues. If anything, participants had a very relaxed attitude toward reporting and having their activities recorded in our specific setting. Many participants disliked the requirement of an additional study phone. A subset reported frustration with the sporadic technical issues (eg, unable to connect the brush to the phone app, lag in establishing connection) and short battery life of the study phone. Comments to the open-ended exit interviews revealed that participants largely preferred the automaticity of ROBAS to the tedium of maintaining a daily diary log of their brushing sessions.

Our design objective was to develop a human activity recognition system that would weave into the fabric of everyday life to extend the reach and continuity of health care. To that end, we leveraged everyday devices (electronic toothbrushes and smartphones) and habitual behaviors (brushing) to passively monitor oral self-care practices in the lived environment. Tools, like ROBAS, that introduce passive measurement into the delivery of dental care have considerable potential in allowing actionable insights on actual oral self-care practices in the home setting. The objective data gathered by the sensing technology could eventually drive computationally driven, adaptive behavioral nudges that automatically adjust to the individuals’ changing behavior, history, and environmental contexts [29]. Temporal changes in the data sets would help determine who engages in the interventions, how they engage, and factors that promote engagement. Furthermore, patterns around self-care behaviors would allow care providers and health systems to proactively identify and focus on those most at risk to ultimately mitigate the high burden and costs of dental disease.

We feel that ROBAS is the closest alternative to direct observation of brushing behaviors and a valuable new tool for researchers interested in investigating or measuring oral self-care behavior. Ultimately, the usability of the system will determine user adoption. Based on our study results, several technological improvements to the ROBAS prototype have been carried out. These include an updated smart toothbrush Bluetooth software development kit (SDK), the addition of a gyroscope within the smart toothbrush, and more robust mechanisms to handle and detect the Bluetooth connection challenges on Android smartphones. The use of a dedicated study phone, a perceived burden in the ROBAS testing, has been abandoned in favor of a Bring Your Own Device (BYOD) model in which participants install the mCerebrum app on their own mobile phone. Although this introduces a technical complexity in that participants are required to provide a phone that is fit for purpose, the pragmatic move greatly increases the pool of participants available for follow-up studies and decreases the provisioning costs (ie, costs of a dedicated phone) and supply and training issues (ie, delivering the devices to the participants and training them).

The promising results notwithstanding, our study had some limitations. As discussed above, some participants may have used the brushing durations displayed by the ROBAS app instead of independently measuring brushing duration using a separate stopwatch, artificially increasing the concordance between the diaries and the ROBAS data. Additionally, selection bias influences the levels of measurement agreement found in this study. Although we recruited from the community, study participants had to opt into this study, attend an orientation visit, complete the study protocol, and return for an exit interview. This requirement may have led to a sample with higher levels of success in the use of ROBAS and accuracy in recording the diaries than the general population. 

### Conclusion

Based on our study findings, ROBAS has a high criterion validity for measuring oral health behaviors. It can accurately and reliably monitor brushing behaviors in the home setting for extended periods. Unobtrusive data collection through ROBAS sets the stage for automated coaching and optimization of oral self-care practices for each individual across the population.

## Appendix

Multimedia Appendix 1 Discrepancies in number of sessions recorded (diary – ROBAS) versus number of glitches reported in diary. ROBAS: Remote Oral Behaviors Assessment System.

## Abbreviations

BLE: Bluetooth Low Energy
BYOD: Bring Your Own Device
MAPE: mean absolute percent error
OHB: oral hygiene behavior(s)
ROBAS: Remote Oral Behaviors Assessment System
SDK: software development kit
