# Supplementary figures and images for: A Scalable System for Passively Monitoring Oral Health Behaviors Using Electronic Toothbrushes in the Home Setting: Development and Feasibility Study
Source: JMIR Mhealth Uhealth. 2020 Jun 24;8(6):e17347. doi: 10.2196/17347 (PMC7380983; doi:10.2196/17347)

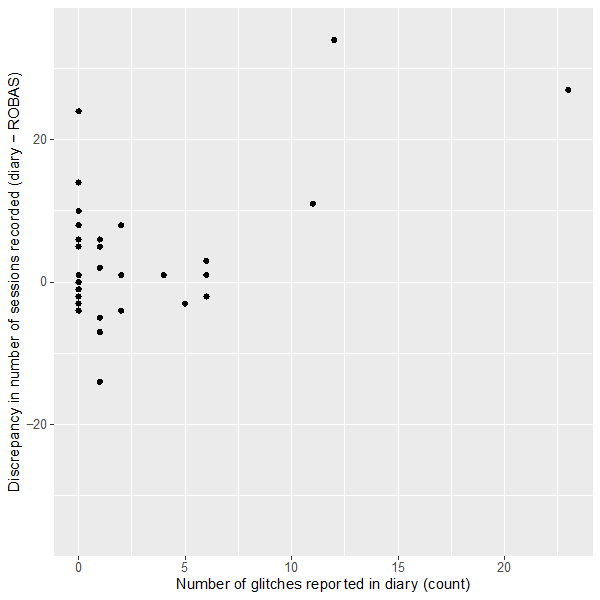

Supplement: Multimedia Appendix 1 [file mhealth_v8i6e17347_app1.png]
